# Cognitive Perception among Post-Chemotherapy, Non-Chemotherapy Breast Cancer Survivors and Non-Cancer

**DOI:** 10.31557/APJCP.2021.22.6.1775

**Published:** 2021-06

**Authors:** Hilman Syarif, Agung Waluyo, Yati Afiyanti

**Affiliations:** 1 *Department of Medical Surgical Nursing, Faculty of Nursing, Universitas Syiah Kuala, Banda Aceh, Indonesia. *; 2 *Medical Surgical Nursing Department, Faculty of Nursing, Universitas Indonesia, Depok, East Java, Indonesia. *; 3 *Maternity Department, Faculty of Nursing, Universitas Indonesia, Depok, East Java. Indonesia. *

**Keywords:** Breast cancer, chemotherapy, cognitive impairment, survivor

## Abstract

**Objective:**

This study aims to compare the cognitive function perceived by post-chemotherapy breast cancer survivors, breast cancer survivors without chemotherapy, and non-cancer woman patients.

**Methods:**

This study was conducted by a descriptive comparative method. The respondents consisted of 82 survivors of post-chemotherapy breast cancer, 81 non-chemotherapy breast cancer survivors, and 80 non-cancer woman patients who were recruited using consecutive sampling. The data were collected from October until December 2017 by using a FACT-Cog version 3 questionnaire. Data analysis was performed by using a comparative test of Kruskal-Wallis followed by a Mann-Whitney posthoc analysis.

**Results:**

The results showed that the median of cognitive function perception in breast cancer survivors post-chemotherapy, non-chemotherapy, and non-cancerous women are 94 (52-122), 113 (53-130), and 121 (69-132), respectively. Mann-Whitney’s post-hoc analysis showed a significantly different perception of cognitive function between post-chemotherapy survivors and non-chemotherapy survivors; also between post-chemotherapy survivors and non-cancer women, and between non-chemotherapy survivors with non-cancer women.

**Conclusion:**

Perceived cognitive impairment, comments from others, perceived cognitive abilities, and effects on quality of life in breast cancer survivors who received chemotherapy are significantly different as compared to the survivor group without chemotherapy and non-cancerous healthy women.

## Introduction

One particular thing that concerned breast cancer survivors are cognitive dysfunction, commonly known as chemobrain. This phenomenon has become the main research focus on cancer survivors by health care providers (Selamat et al., 2014). Chemobrain refers to a common mental foggy in patients experienced with cancer and its treatment. These conditions include extensive symptoms such as short-term memory loss, difficulty in thinking and maintaining concentration, multitasking disorders, and other subtle cognitive changes (Argyriou et al., 2011; Asher and Myers, 2015). In general, the study showed that cognitive problems caused negative effects and it dramatically affected patients’ function, quality of life, and community integration (Argyriou et al., 2011; Janelsins et al., 2014; Vardy et al., 2008). 

Chemobrain has its consequences on various life dimensions. There were reported effects such as of 75% of patients had reduced working performance, 58% of patients had to use a compensation strategy, 50% of patients were frustrated, and 33% of patients had adverse effects on family relationships (Ah et al., 2013). In general, the research highlighted survivors of cancer who reported difficulties in job activity as well as maintaining a functional level of ability, thus requiring increased mental support (Ahles et al., 2012). Chemobrain experienced by the respondents caused frustration, influenced confidence, and social relationships caused difficulty in working and adaptability using compensation strategies (Janelsins et al., 2011).

The prevalence of cognitive dysfunction varied greatly in the literature (Asher and Myers, 2015). There are about 75% of cancer survivors had experienced cognitive impairment during treatment and about 15%-35% of survivors of cancer had cognitive problems within months to years after treatment (Lai et al., 2009; Wefel et al., 2008). A total of 16%-75% of survivors of breast cancer had experienced cognitive impairment during treatment compared with 4%-11% in the control group (Wefel et al., 2008; Cerulla et al., 2019). The variation of prevalence inhibits accurate measurement of incidence of cognitive dysfunction, including lack of pre-treatment cognitive assessment, research population differences, lack of standardized tools, and neuropsychological barriers. Chemobrain measurements were also hampered by differences in inclusion criteria, cognitive measurement times, and variations in comparison groups (Asher and Myers, 2015). Additionally, the type of tumor, the stage of the disease, the treatment used, and the medical comorbidities also affected (Argyriou et al., 2011; Munir et al., 2010). Cultural factors also influenced the survivors’ expressions. It showed that Asian women are less familiar with the chemobrain phenomena than Western women (Selamat et al., 2014). Western cultures were emotionally more expressive in emotion (Munir et al., 2010). Indonesia has several hospitals that provide chemotherapy services so that the number of breast cancer survivors with chemotherapy is abundant. The 5-year survival rate of the breast cancer patient who received neoadjuvant chemoradiation, surgery, and then adjuvant chemotherapy was 64.7%, while those who receive operation and continued by chemotherapy and radiotherapy was 72.9% (Boykoff et al., 2009). However, research related to cognitive complaints in postchemotherapy breast cancer survivors is still rare. This study aims to compare the cognitive functions of post-chemotherapy breast cancer survivors, non-chemotherapy breast cancer survivors, and non-cancer women patients using FACT Cog version 3.

## Materials and Methods

*Participant*

This research was conducted by using a comparative descriptive with a cross-sectional design. Data was collected from Fatmawati general hospital in Jakarta, Jakarta Special Capital Region, and Hasan Sadikin general hospital in Bandung from October until December 2017. The study respondents were divided into three groups such as 82 post-chemotherapy breast cancer survivors as group I, 81 survivors of breast cancer without chemotherapy as group II, and 80 non-cancer women as group III. Sampling was performed as a consecutive sampling method. Their post-chemotherapy inclusion should have completed certain criteria, for instance, six cycles of chemotherapy and diagnosed in stage I-III. Whilst, respondents within the non-chemotherapy group should not have received chemotherapy and diagnosed in stage I-III. All respondents were 21-55 years without any psychiatric disorders and neurological disorders.

*Design*

All the data was collected after accepted by the Research Ethics Committee of Faculty of Nursing, Universitas Indonesia (250/UN2.F12.D/HKP.02.04/2017) and permitted by two research hospitals. All study respondents have gained in-depth information regarding the research, showed signs of approval, and signed informed consent before data collection.

The Functional Assessment of Cancer Therapy-cognitive version three (FACT-Cog v3) was used as a questionnaire in this study. It consisted of 37 statement items whereas each statement has a score range from 0 to 4. However, the scoring instructions of the questionnaire are merely allowed 33 statements to be used. Therefore, the minimum score is 0 and the highest score is 132. A higher score indicated a better perception of cognitive function. The questionnaire is identified as four dimensions, such as 1) Perception of cognitive impairment in 18 statements with score 0-72; 2) Comments from others in four statements with score 0-16; 3) Perceptual-cognitive ability in seven statements with score 0-28, and 4) Effect of cognitive change on the quality of life in four states with score 0-16.

The questionnaire has been translated into Indonesian, then it was translated back into English. Furthermore, the measurement of validity, legibility, and reliability test was also performed in this research. In brief, the content of the validity test was demonstrated by three nursing experts, the legibility test was performed on 10 breast cancer survivors, and the reliability test was performed on 30 breast cancer survivors at Fatmawati general hospital in Jakarta. Results showed that all items were valid with a Cronbach alpha score 0.82.

*Data analysis*

Data analysis consisted of univariate analysis and comparative hypothesis test with more than two averages value. Univariate analysis was conducted to identify the mean value, standard deviation, median, maximum, and minimum value of age, perception of total cognitive function, and its components. Also, it was useful to identify the frequency and percentage of education length, employment, and marital status of the respondents. The comparative hypothesis test with more than two averages was performed by using Kruskal-Wallis test which followed by post-hoc analysis using Mann-Whitney.

## Results

The average age of each group is varied. Respondents belong to group I has an average age of about 45.41±6.98 years, group II is 44.63±6.32 years, and group III is 39.05±9.5 years. There are also other various parameters described. In detail, group III has 88.8% respondents with ≤ 12 years of education length, and 65% respondents of employed women, and 100% of the respondent with non-used hormonal therapy. While, in group I, there are 85.3% of respondents are married women and 79.3% of respondents are menopause. The data is represented in Table 1.

Table 2 showed the distribution of respondents’ answers for some statements. Complaints of cognitive impairment are mostly found within respondents in group I whereas all questions are found with a greater percentage

Perception of cognitive function significantly different among the three groups. Mann-Whitney’s posthoc analysis showed a significantly different perception of cognitive function between group I and group II; group I and group III; also group II and group III. The cognitive impairment perception is also significantly different among the three groups. The post-hoc analysis with Mann-Whitney demonstrated a significantly different perception of cognitive impairment between group I and group II, group II and group III, then the group I and group III. Comments received by respondents from others about their cognitive changes significantly different also the three groups. Moreover, the Mann-Whitney post-hoc analysis showed comments from others which significantly different between group I and group II, also group I and group III; while group II and group III are not significantly different. However, there is a significant difference concerning the perception of cognitive abilities among groups. Mann-Whitney’s post-hoc analysis demonstrated significantly different perceptions of cognitive abilities between group I and group II, between group II and group III, then between group I and group III. The effect of this cognitive disorder is significantly different among respondents. Mann-Whitney’s post-hoc analysis showed significant differences between group I and group II, also between group I and group III; while group II and group III are not significantly different (Table 3).

**Table 1:** Distribution of Respondents by Age, Length of Education, Occupation, Marital Status, Hormonal Therapy Usage, and Menopausal Status (n=243)

	Group I [n (%)]	Group II [n (%)]	Group III [n (%)]
Age (year)	45.41±6.98	44.63±6.32	39.05±9.5
Duration of education			
≤12 yrs	62 (75.6)	64 (79)	71(88.8)
>12 yrs	20 (24.4)	17 (21)	9 (11.3)
Employment status			
Employed	38 (46.3)	44 (54.3)	52 (65)
Unemployed	44 (53.7)	37 (45.7)	28 (35)
Marital status			
Single	4 (4.9)	3 (3.7)	7 (8.8)
Widow	8 (9.8)	12 (14.8)	8 (10)
Married	70 (85.3)	66 (81.5)	65 (81.2)
Hormon therapy usage			
Yes	34 (41.4)	12 (14.8)	0 (0)
No	48 (58.6)	69 (85.2)	80 (100)
Menopausal status			
Post-menopause	65 (79.3)	42 (51.8)	18 (22.5)
Pre-menopause	17 (20.7)	39 (48.2)	62 (77.5)

**Table 2:** Distribution of Respondents’s Answer for Each Statement

	Group I [n(%)]		Group II [n(%)]		Group III [n(%)]	
	Several times a day	Nearly every day	Several times a day	Nearly every day	Several times a day	Nearly every day
Trouble forming thoughts	2 (2.4)	6 (7.3)	0 (0)	2 (2.5)	1 (1.3)	2 (2.5)
Thinking has been slow	6 (7.3)	21 (25.6)	1 (1.2)	1 (1.2)	0 (0)	0 (0)
Trouble concentrate	3 (3.7)	24 (29.3)	1 (1.2)	2 (2.5)	1 (1.3)	3 (3.8)
Trouble remembering where put things, like keys or wallet	3 (3.7)	26 (31.7)	0 (0)	2 (2.5)	0 (0)	3 (3.8)
Trouble remembering new information	3 (3.7)	21 (25.6)	1 (1.2)	7 (8.6)	0 (0)	3 (3.8)
Trouble recalling the name of an object while talking	1 (1.2)	7 (8.5)	0 (0)	1 (1.2)	0 (0)	1 (1.3)
Trouble finding the right word(s) to express myself	1 (1.2)	6 (7.3)	0 (0)	0 (0)	0 (0)	0 (0)
Trouble saying what I mean in conversations with others	3 (3.7)	8 (9.8)	0 (0)	2 (2.5)	1 (1.3)	0 (0)
To work really hard to pay attention	10 (12.2)	4 (4.9)	1 (1.2)	0 (0)	2 (2.5)	4 (5.0)
Forgotten names of people soon after being introduced	13 (15.9)	2 (2.4)	5 (6.2)	7 (8.6)	2 (2.5)	6 (7.5)
To work harder than usual to keep track of activities	14 (17.1)	7 (8.5)	1 (1.2)	3 (3.7)	3 (3.8)	3 (3.8)
Thinking has been slower than usual	5 (6.1)	6 (7.3)	1 (1.2)	2 (2.5)	0 (0)	4 (5.0)
	Not al all	A little bit	Not al all	A little bit	Not al all	A little bit
Ability to concentrate	6 (7.3)	15 (18.3)	1 (1.2)	1 (1.2)	0 (0)	4 (5.0)
Ability to bring to mind words while talking to someone	8 (9.8)	31 (37.8)	0 (0)	5 (6.2)	0 (0)	0 (0)
Ability to remember things	1 (1.2)	21 (25.6)	0 (0)	3 (3.8)	0 (0)	3 (3.8)
Ability to remember to do things	1 (1.2)	5 (6.1)	0 (0)	3 (3.7)	0 (0)	2 (2.5)
Mind is as sharp as it has always been	2 (2.4)	20 (24.4)	0 (0)	6 (7.4)	2 (2.5)	4 (5.0)
Memory is as good as it has always been	3 (3.7)	14 (17.1)	1 (1.2)	5 (6.2)	0 (0)	4 (5.0)
	Very much	Quite a bit	Very much	Quite a bit	Very much	Quite a bit
These problems have interfered the quality of life	2 (2.4)	2 (2.4)	0 (0)	0 (0)	0 (0)	0 (0)

**Table 3:** Comparison Perception of Cognitive Function and Components between Groups (n=243)

	Median (min-maks)	Mean rank	X^2^	p value
Perception of cognitive function				
Group I	94 (52-122)	70.14	76.224	<0.001
Group II	113(53-130)	132.07		
Group III	121 (69-132)	164.96		
Kruskal-Wallis test. Mann-Whitney test: Group I vs Group II p=0.000; Group I vs Group III p=0.000; Group II vs Group III p=0.000				
Perception of cognitive impairment				
Group I	49 (20-68)	74.02	67.408	<0.001
Group II	60 (19-72)	129.35		
Group III	68 (30-72)	163.74		
Kruskal-Wallis test. Mann-Whitney test: Group I vs Group II p=0.000; Group I vs Group III p=0.000; Group II vs Group III p=0.000				
Comment from others				
Group I	16 (8-16)	104.05	13.218	0.001
Group II	16 (10-16)	125.19		
Group III	16 (10-16)	137.17		
Kruskal-Wallis test. Mann-Whitney test: Group I vs Group II p=0.018; Group I vs Group III p=0.001; Group II vs Group III p=0.110				
Perception of cognitive ability				
Group I	17 (5-26)	77.15	56.262	<0.001
Group II	21 (4-28)	131.8		
Group III	23 (8-28)	158.05		
Kruskal-Wallis test. Mann-Whitney test: Group I vs Group II p=0.000; Group I vs Group III p=0.000; Group II vs Group III p=0.005.				
The effect of cognitive impairment on quality of life				
Group I	14 (6-16)	83.63	45.829	<0.001
Group II	16 (11-16)	137.31		
Group III	16 (9-16)	145.83		
Kruskal-Wallis test. Mann-Whitney test: Group I vs Group II p= 0.000; Group I vs Group III p=0.000; Group II vs Group III p=0.192				

## Discussion

The median score of perception of cognitive function in breast cancer survivors post-chemotherapy, without chemotherapy, and non-cancerous women was 94 (52-122), 113 (53-130), and 121 (69-132), respectively. In line with research of Cheung et al. (2012a), respondents who received chemotherapy and 33 questions FACT Cog v3 exhibited median cognitive function perceptual score 110, while those who did not receive chemotherapy had a score 124 (Hirokawa et al., 2004). These results are different from research of Cheung et al, who found 127.6 (18.1) of an average score of FACT Cog v3 in survivors of breast cancer who received chemotherapy and median 132 (Cheung et al., 2014; Hendrik et al., 2012). This difference might occur because they used 37 statements, so the resulted mean score became higher.

Perceptions of cognitive function significantly different among the three groups of respondents. This difference in perception occurred due to different experiences regarding cognitive function changes within three groups, which was clearly experienced by groups post-chemotherapy. This could be triggered directly by chemotherapy. The direct neurotoxic effect of chemotherapy is demonstrated a clear hypothesis for the cognitive impairment etiology in chemobrain. Cognitive changes mechanism happened after chemotherapy are tissue trauma and inflammation that could trigger systemic inflammation, then cross the blood-brain barrier (BBB), and further disrupt the central nervous system (Cheung et al., 2012a; Bajic et al., 2018). In addition, chemotherapy drugs in significant amounts worked through anti-neoplasmic activity that resulted in oxidative stress on malignant tissues such as in the brain, other organs, and systems (Cheung et al., 2014; Zhang et al., 2018). Perception of cognitive function is also significantly different between survivors group without chemotherapy with non-cancerous female patients. In addition to the direct effects of chemotherapy, cognitive decline was also influenced by other factors such as hormone therapy and stress (Janelsins et al., 2011; Cheung et al., 2014). Approximately 14.8% of respondents in the survivor’s group without chemotherapy were using hormone therapy. That therapy hurt cognitive function while used single or combined with other therapy (Janelsins et al., 2011; Ganz et al., 2013). The average stress score in the survivors group without chemotherapy was 12.65, and in the non-cancerous women group, the score was 10.28.

The results showed a significant difference in perception of cognitive impairment by post-chemotherapy breast cancer survivors as compared to those who did not receive chemotherapy. In line with the study was conducted in China, the cognitive impairment perceived by breast cancer respondents given chemotherapy significantly different from respondents without chemotherapy (median 63 vs 68; p < .001) (Hirokawa et al., 2004). Another research also explained that cognitive impairment perceived by survivors of breast cancer showed a mean score of 56.8 (11.2), while the minimum and maximum of 26-72 (Gaman et al., 2016). Chemotherapy might decrease cognitive function, which leads to a difference in perception of cognitive impairment between post-chemotherapy survivors and those without chemotherapy. This perception depicted the experiences of respondents. Many post-chemotherapy groups reported difficulty in coming up with ideas, slowing thinking processes, difficulty in maintaining concentration, and remembering new information. Furthermore, they ought to try harder than usual to meet work expectations. In line with research the majority of their respondents reported a loss of memory, difficulty in decision making, and verbal communication impairment after receiving chemotherapy (Bender et al., 2001; Shen et al., 2019). Such experiences were rarely encountered in non-cancer sick women, so they have a good perception of their cognitive function.

The study also showed that there were significant differences regarding comments from family members, friends, and colleagues about the fact of decrease in cognitive ability on breast cancer survivors post-chemotherapy with those non-chemotherapy group (p = .018), but there was no significant difference between survivors non-chemotherapy group and non-cancer group (p = .110). This is in line with the research stated that breast cancer patients with chemotherapy had a median comment score of 16 (maximum value 16) and also 3 months later with no score changes (Hendrik et al., 2012). Another study showed a mean score of 15.1 ±1.4 in a comment from others’ section, with a minimum to maximum score ranged from 11-16 (Gaman et al., 2016). Others felt changes that have occurred due to cognitive impairment experienced by the survivors, so they make comments about that. The family and friends’ reaction after the appearance of symptoms varied from apathy to support. In this study, comments from family members and friends rarely point out to the problems, because of culture in Indonesia less expressive on making a comment to others. In line with the several studies that exhibited chemobrain caused confusion within family and friends circles, especially children (Joly et al., 2012). Sometimes an adult person also did not understand any cognitive changes. Family members sometimes make a comparison between their cognitive impairment with dementia (Cheung et al., 2012b). The negative impact on family came from the expectations of the respondents themselves because they were incapable of performing their role as a mother and wife in the family due to cognitive decline (Bender et al., 2001; Shin et al., 2018).

Other information gained from this study showed a significant difference in perceived cognitive abilities between breast cancer survivors post-chemotherapy and non-chemotherapy (p < .001), as well as the non-cancerous women (p < .001). In line with the research, the cognitive ability score perceived by breast cancer patients with chemotherapy resulted in median score of 21 (maximum score is 28), while those without chemotherapy had score of 25 (Hirokawa et al., 2004). Another research, the mean of cognitive ability score was perceived by survivors was 18.2±5.2, the minimum to maximum value ranged from 5 to 27 (Gaman et al., 2016). Survivors of breast cancer post-chemotherapy felt decreased cognitive abilities. Approximately, 7.3% of respondents said that their ability to concentrate is in “not at all” category, while 18.3% of respondents is in “a little bit” category. It was identified by survivors post-chemotherapy group as cognitive decline experience in daily life after received chemotherapy regiment. It’s in also in line with the study whose participants reported problems with their memory as most of the respondents identified it as their primary concern. The most frequently reported memory issues included an inability to remember important names, events, and appointment schedules (Janelsins et al., 2011). Respondents in the study also reported low ability in concentration, confused, and a decline in clear thought (Cheung et al., 2012a; Ng et al., 2017).

The effects on quality of life also differed significantly between breast cancer survivors who received chemotherapy with those without chemotherapy, as well as between breast cancer survivors who underwent chemotherapy with non-cancerous women. The effect showed the quality of life with median score of 15 (maximum score 16), which then decreased to 14 after 3 months duration (Hendrik et al., 2012). Another study showed the effect on quality of life score with an average value of 11.7 (4.2), the minimum to maximum value ranged from 0-16 (Gaman et al., 2016). In line with research on 74 breast cancer survivors who experienced with post-chemotherapy side effects showed that cognitive impairment became a major problem for them (Cheung et al., 2012b). Survivors reported decreased quality of life and reduced daily functioning as a result of chemobrain. The cognitive decline could reduce the quality of life of breast cancer survivors because this problem might lead to limitations in performing work, doing social interactions, and conducting favored activities. Next, affected their quality of life (Cheung et al., 2012a). However, the degree of complaints about the decline of their quality of life different from research in western countries, because survivors in Indonesia are less expressive or less vocal about their conditions. In line with another study, survivors in Asian country less vocal if compared with survivors in west countries (Selamat et al., 2014).

Perceived cognitive impairment, comments from others, perceived cognitive abilities, and effects on quality of life in breast cancer survivors who received chemotherapy are significantly different as compared to the survivor group without chemotherapy and non-cancerous healthy women. The further cognitive abilities assessment is expected to be performed in breast cancer survivors who will receive a chemotherapy treatment. Therefore, it is essential for nurses to consider the cognitive ability assessment for survivors in order to develop the holistic nursing care plan.

## Author Contribution Statement

All authors worked on data analysis and manuscript preparation. The final manuscript has already approved by all authors.
